# Diagnostic and prognostic impact of cytokeratin 18 expression in human tumors: a tissue microarray study on 11,952 tumors

**DOI:** 10.1186/s10020-021-00274-7

**Published:** 2021-02-15

**Authors:** Anne Menz, Timo Weitbrecht, Natalia Gorbokon, Franziska Büscheck, Andreas M. Luebke, Martina Kluth, Claudia Hube-Magg, Andrea Hinsch, Doris Höflmayer, Sören Weidemann, Christoph Fraune, Katharina Möller, Christian Bernreuther, Patrick Lebok, Till Clauditz, Guido Sauter, Ria Uhlig, Waldemar Wilczak, Stefan Steurer, Sarah Minner, Eike Burandt, Rainer Krech, David Dum, Till Krech, Andreas Marx, Ronald Simon

**Affiliations:** 1 Institute of Pathology, University Medical Center Hamburg-Eppendorf, Martinistr. 52, 20246 Hamburg, Germany; 2 Institute of Pathology, Clinical Center Osnabrueck, Osnabrueck, Germany; 3 Department of Pathology, Academic Hospital Fuerth, Fuerth, Germany

**Keywords:** Cytokeratin 18 (CK18), Tissue microarray, Immunohistochemistry

## Abstract

**Background:**

Cytokeratin 18 (CK18) is an intermediate filament protein of the cytokeratin acidic type I group and is primarily expressed in single-layered or “simple” epithelial tissues and carcinomas of different origin.

**Methods:**

To systematically determine CK18 expression in normal and cancerous tissues, 11,952 tumor samples from 115 different tumor types and subtypes (including carcinomas, mesenchymal and biphasic tumors) as well as 608 samples of 76 different normal tissue types were analyzed by immunohistochemistry in a tissue microarray format.

**Results:**

CK18 was expressed in normal epithelial cells of most organs but absent in normal squamous epithelium. At least an occasional weak CK18 positivity was seen in 90 of 115 (78.3%) tumor types. Wide-spread CK18 positivity was seen in 37 (31.9%) of tumor entities, including adenocarcinomas of the lung, prostate, colon and pancreas as well as ovarian cancer. Tumor categories with variable CK18 immunostaining included cancer types arising from CK18 positive precursor cells but show CK18 downregulation in a fraction of cases, tumor types arising from CK18 negative precursor cells occasionally exhibiting CK18 neo-expression, tumors derived from normal tissues with variable CK18 expression, and tumors with a mixed differentiation. CK18 downregulation was for example seen in renal cell cancers and breast cancers, whereas CK18 neo-expression was found in squamous cell carcinomas of various origins. Down-regulation of CK18 in invasive breast carcinomas of no special type and clear cell renal cell carcinomas (ccRCC) was related to adverse tumor features in both tumors (p ≤ 0.0001) and poor patient prognosis in ccRCC (p = 0.0088). Up-regulation of CK18 in squamous cell carcinomas was linked to high grade and lymph node metastasis (p < 0.05). In summary, CK18 is consistently expressed in various epithelial cancers, especially adenocarcinomas.

**Conclusions:**

Down-regulation or loss of CK18 expression in cancers arising from CK18 positive tissues as well as CK18 neo-expression in cancers originating from CK18 negative tissues is linked to cancer progression and may reflect tumor dedifferentiation.

## Introduction

Cytokeratin 18 (CK18) belongs to the cytokeratin acidic type I group (CK9-CK12) and is encoded by a gene located at chromosome 12q13 (Moll et al. 1982; Waseem et al. 1990). CK18 is an intermediate filament protein that forms heteropolymers with its co-expressed complementary type II keratin partner CK8, which assembles into keratin filaments—the major structural component in the cytoplasm of epithelial cells (Moll et al. 1982; Fuchs and Weber 1994). CK18 is primarily expressed in single-layered or “simple” epithelial tissues of, for example, the liver, kidney, breast, prostate, gastrointestinal tract as well as in cancers arising from CK18 positive epithelial cells (Oshima et al. 1996; Cajaiba et al. 2006; Skinnider et al. 2005; Faridi et al. 2018). Beside the important structural function, CK18 was also shown to play a role in apoptosis (Caulin et al. (2000); Gilbert et al. 2001), cell cycle progression (Galarneau et al. 2007), and cancer-related signaling pathways. For example, CK18 hypoglycosylation is linked to decreased Akt1 kinase activity and reduced cell survival (Rotty et al. 2010). CK18 upregulation was described to be associated with decreased cell motility and invasiveness via the Wnt-pathway (Yee et al. 2010), and CK18 may be involved in the control of the ERK1/2-MAPK pathway (Zhang et al. 2006; Gilbert et al. 2004).

In surgical pathology, CK18 is used as an epithelial marker to identify CK18 positive adenocarcinomas that arise from different CK18 positive normal epithelia (Oshima et al. 1996; Weng et al. 2012). CK18 expression was also suggested as a potential prognostic marker. For example, decreased CK18 expression was found to be related to tumor progression in breast and colorectal cancers (Woelfle et al. 2004; Knosel et al. 2006). Elevated CK18 protein levels were found to be associated with unfavorable tumor features in oral and esophageal squamous cell carcinomas (Makino et al. 2009; Fillies et al. 2006) as well as in non-small cell lung cancers (Zhang et al. 2016). CK18 antibodies have been used as diagnostic cancer markers for more than thirty years (Oshima et al. 1996). However, the literature on the prevalence of CK18 expression is controversial for many cancers (Walker et al. 2007; Shao et al. 2012; Bartek et al. 1991; Malzahn et al. 1998; Young et al. 2002; Lyda and Weiss 2000; Broers et al. 1988; Hsu et al. 2010; Ueda et al. 1993; Lam et al. 2001; Moll et al. 1993; Levy et al. 1992; Notohara et al. 2000; Akiba et al. 2016; Shimonishi et al. 2000; Yoshikawa et al. 1998; Sinard 1999; Poniecka and Alexis 1999; Balm et al. 1996; Nanda et al. 2012; Agaimy et al. 2012; Miettinen and Fetsch 2000; Raju 1988; Chen et al. 2011; Ishida et al. 2017; Nhung et al. 1999; Safadi et al. 2019). For example, CK18 positivity has been described in 30% to 100% of oral squamous cell carcinomas (Nanda et al. 2012; Safadi et al. 2019), 0% to 100% of non-small cell lung cancers (Chen et al. 2011; Nhung et al. 1999), and 0% to 43% of esophageal squamous cell carcinomas (Makino et al. 2009; Ishida et al. 2017). These conflicting data are likely to be caused by the use of different antibodies, immunostaining protocols, and criteria to determine CK18 positivity in these studies.

To better understand the prevalence and significance of CK18 expression in cancer, a comprehensive study analyzing a large number of neoplastic and non-neoplastic tissues under highly standardized conditions is needed. Therefore, CK18 expression was analyzed in more than 14,000 tumor tissue samples from 115 different tumor types and subtypes as well as 76 non-neoplastic tissue categories by immunohistochemistry (IHC) in a tissue microarray (TMA) format in this study.

## Materials and methods

### Tissue microarrays (TMAs)

Our normal tissue TMA was composed of 8 samples from 8 different donors for each of 76 different normal tissue types (608 samples on one slide). The cancer TMAs contained a total of 14,579 primary tumors from 115 tumor types and subtypes. Detailed histopathological data on grade, pT and pN status were available from 4191 cancers (breast, kidney, bladder, various kinds of squamous cell carcinoma). Clinical follow up data were available from 1178 breast cancer and 847 kidney cancer patients with a median follow-up time of 49/39 months (range 1–88/1–250). The composition of both normal and cancer TMAs is described in detail in the results section. All samples were from the archives of the Institutes of Pathology, University Hospital of Hamburg, Germany, the Institute of Pathology, Clinical Center Osnabrueck, Germany, and Department of Pathology, Academic Hospital Fuerth, Germany. Tissues were fixed in 4% buffered formalin and then embedded in paraffin. The TMA manufacturing process was described earlier in detail (Dancau et al. 2016; Kononen et al. 1998). In brief, one tissue spot (diameter: 0.6 mm) was transmitted from a cancer containing donor block (≥ 70% cancer cells) in an empty recipient paraffin block. The use of archived remnants of diagnostic tissues for manufacturing of TMAs and their analysis for research purposes as well as patient data analysis has been approved by local laws (HmbKHG, §12) and by the local ethics committee (Ethics commission Hamburg, WF-049/09). All work has been carried out in compliance with the Helsinki Declaration.

### Immunohistochemistry

Freshly cut TMA sections were immunostained on one day and in one experiment. Slides were deparaffinized and exposed to heat-induced antigen retrieval for 5 min in an autoclave at 121 °C in pH 7.8 buffer. Primary antibody specific for CK18 (mouse monoclonal, MSVA-118, MS Validated Antibodies, GmbH, Hamburg, Germany) was applied at 37 °C for 60 min at a dilution of 1:300. Bound antibody was then visualized using the EnVision Kit (Agilent, CA, USA) according to the manufacturer’s directions. For tumor tissues, the percentage of positive neoplastic cells was estimated, and the staining intensity was semiquantitatively recorded (0, 1 +, 2 +, 3 +). For statistical analyses, the staining results were categorized into four groups. Tumors without any staining were considered negative. Tumors with 1 + staining intensity in ≤ 70% of cells and 2 + intensity in ≤ 30% of cells were considered weakly positive. Tumors with 1 + staining intensity in  > 70% of cells, 2 + intensity in 31–70%, or 3 + intensity in ≤ 30% were considered moderately positive. Tumors with 2 + intensity in > 70% or 3 + intensity in > 30% of cells werde considered strongly positive.

### Statistics

Statistical calculations were performed with JMP 14 software (SAS Institute Inc., NC, USA). Contingency tables and the chi^2^-test were performed to search for associations between CK18 and tumor phenotype. Survival curves were calculated according to Kaplan–Meier. The Log-Rank test was applied to detect significant differences between groups.

## Results

### Technical issues

A total of 11,952 (82.0%) of 14,579 tumor samples were interpretable in our TMA analysis. The remaining 2627 (18.0%) samples were not analyzable due to the lack of unequivocal tumor cells or loss of the tissue spot during the technical procedures. On the normal tissue TMA, a sufficient number of samples was always interpretable per tissue to determine the CK18 expression.

### CK18 in normal tissues

**Fig. 1:**
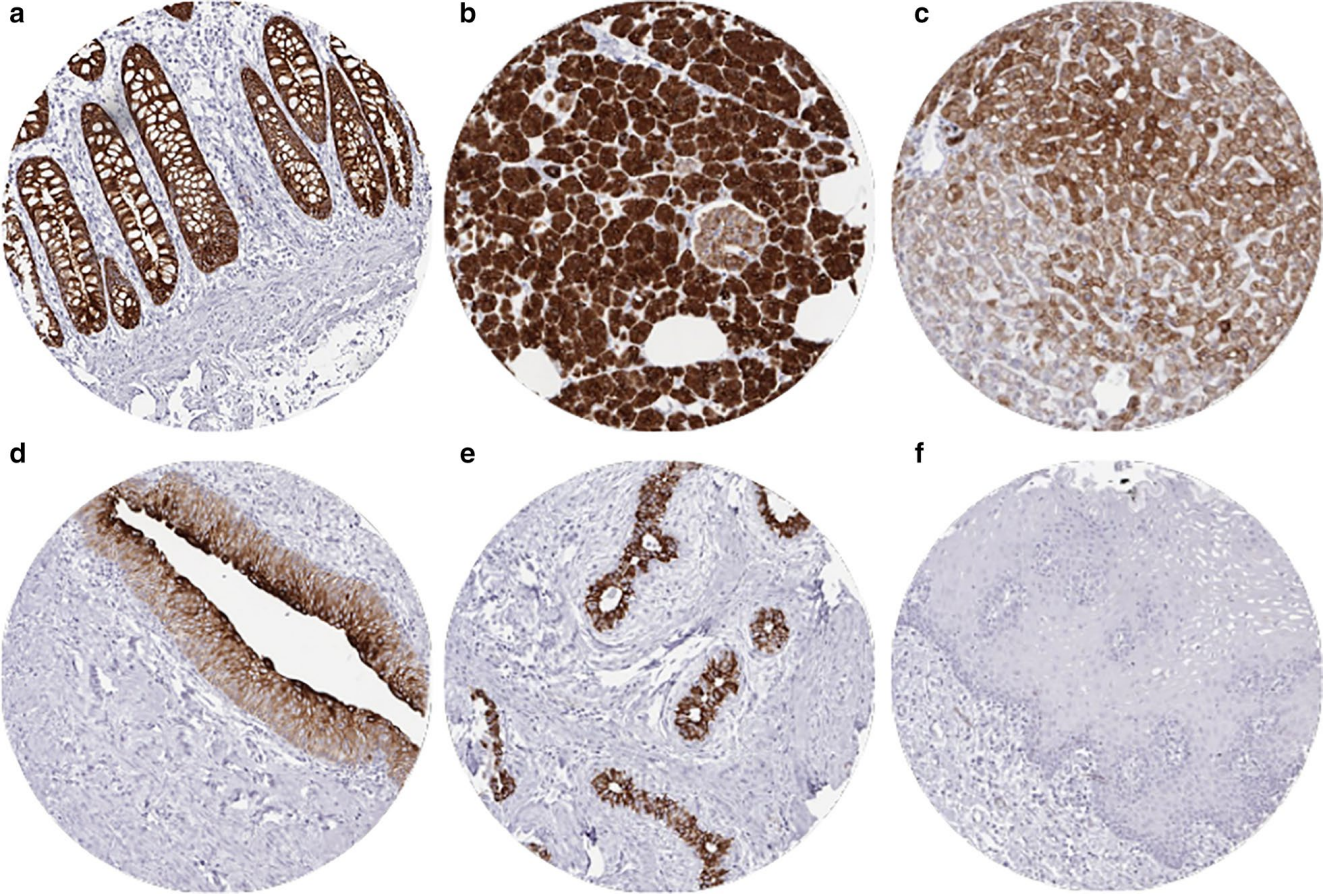
Cytokeratin 18 (CK18) expression in normal tissues. The images show strong CK18 staining in epithelial cells from rectum (**a**) and pancreas (**b**), a zonal staining variability in the liver (**c**), strongly positive umbrella cells and a gradually decreasing staining intensity from superficial to basal urothelial cells in the bladder (**d**), strong positivity in acinus cells but absent staining in basal cells of the breast epithelium (**e**) and a complete lack of staining in squamous epithelium of the oral mucosa (**f**)

CK18 was highly expressed in epithelial cells of the stomach (except parietal cells), duodenum, ileum, appendix, colon, rectum (Fig. 1a), gall bladder, pancreas (weaker staining in Islet cells than in acinus cells; Fig. 1b), endometrium, endocervix, alveolar cells of the lung, cytotrophoblast and syncytiotrophoblast of the placenta, and in all cells of the adenohypophysis (variable staining intensity). Liver tissue exhibited a zonal variability in hepatocyte staining ranging from negative to strongly positive (Fig. 1c). Bile ducts were always positive. Urothelium of the kidney and urinary bladder showed a strong staining in umbrella cells and a gradually decreasing staining intensity from superficial to basal urothelial cells (Fig. 1d). Salivary glands showed strong staining of serous and mucinous cells but somewhat weaker positivity in excretion ducts, especially in large ones. Some ducts only showed few positive cells or complete CK18 negativity. In the kidney, proximal and distal tubuli as well as collecting ducts were CK18 positive. In the ovary, follicular cells and follicular cysts stained positive as well as some cells of the corpus luteum. A strong positive staining of glandular cells with weaker and probably absent staining in basal cells was seen in prostate, respiratory mucosa of bronchus and paranasal sinuses, epididymis, seminal vesicle, and breast glands (Fig. 1e). In lymph nodes, tonsil, spleen, and thymus delicate fibrillar staining caused by CK18 positive fibroblastic reticulum occurred mainly in the interfollicular area. In the thymus, some cellular components of Hassal bodies were CK18 positive, and merkel cells in the skin and hair follicles were CK18 positive. CK18 was absent in all mesenchymal tissues, the stroma of the ovary, posterior lobe of the pituitary gland, brain, bone marrow, lymph nodes, spleen and lymphocytes in tonsil and thymus. Staining was also negative in all squamous epithelia from esophagus, skin, lip, oral cavity (Fig. 1f), tonsil, and anal canal, hair follicles, sebaceous glands, testis (except some weak staining in some Sertoli cells in 2 of 8 samples), adrenal gland (except some cells with weak staining in 1/8 samples), aorta, heart, striated muscle, skeletal muscle, myometrium, muscular wall of the gastrointestinal tract, kidney pelvis, and the urinary bladder, corpus spongiosum of the penis, bone marrow.

### CK18 in neoplastic tissues

**Fig. 2:**
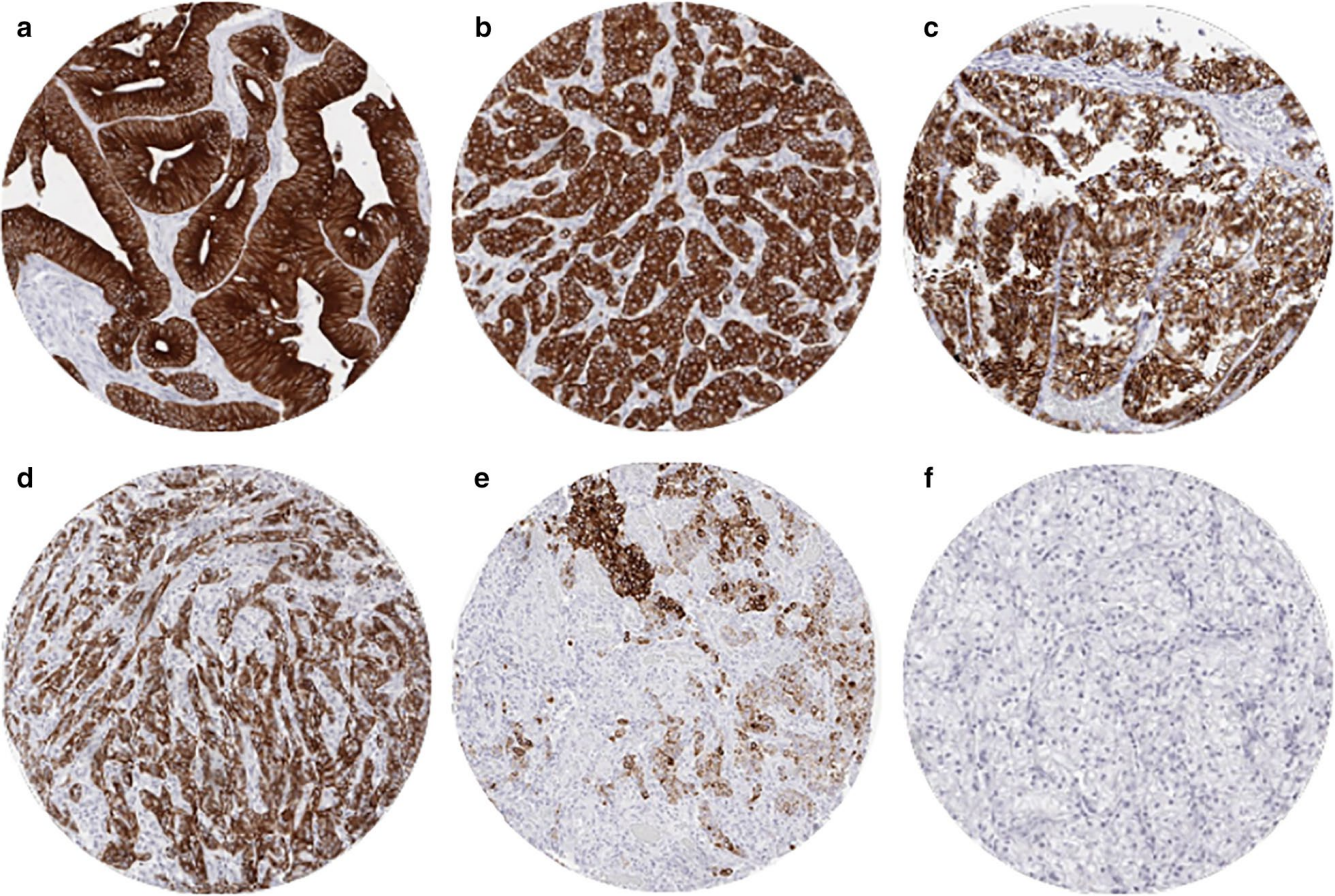
Cytokeratin 18 (CK18) expression in tumors. The images show diffuse strong CK18 staining in a colorectal carcinoma (**a**), an invasive breast carcinoma of no special type (**b**), a clear cell carcinoma of the kidney (**c**), and a squamous cell carcinoma of the cervix uteri (**d**). CK18 immunostaining is focal in a squamous cell carcinoma of the larynx (**e**) and absent in another renal cell  clear cell carcinoma (**f**)

**Fig. 3:**
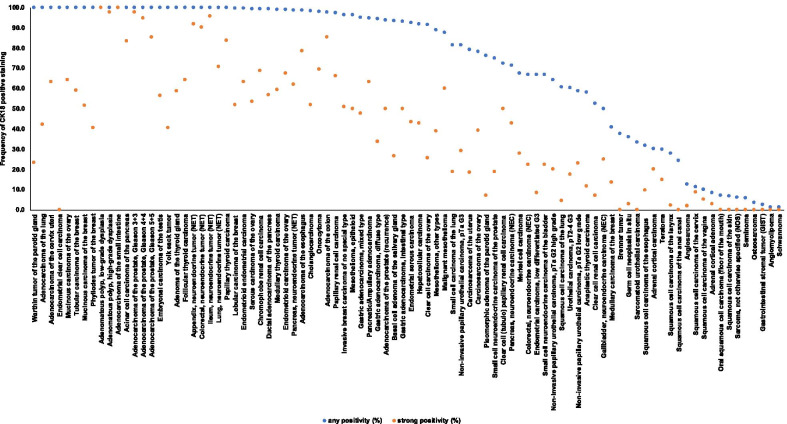
Ranking order of Cytokeratin 18 (CK18) immunostaining in cancers. Both the frequency of positive cases (blue dots) and the frequency of strongly positive cases (orange dots) are shown. The conspicuously low rate of strongly positive Whartin tumors is due to the fact, that only basal cells react with CK18 resulting in a low overall percentage of positive cells. 25 additional tumor entities without any CK18 positive cases are not shown due to space restrictions

Cytoplasmic immunostaining was observed in 9098 (76.1%) of 11,952 analyzable tumors, including 45.0% with strong, 16.5% with moderate, and 14.6% with weak staining intensity. At least an occasional weak CK18 positivity was detected in 90 of 115 (78.3%) different tumor types and tumor subtypes and 78 (67.8%) tumor types and tumor subtypes had at least one tumor exhibiting strong positivity. Representative images of CK18 positive tumors are shown in Fig. 2. The highest frequencies of CK18 positivity were seen in adenocarcinomas of the lung, cervix uteri, small intestine, prostate, and pancreas, some breast cancer and thyroid cancer subtypes, and most of all neuroendocrine tumors and carcinomas. A detailed description of the immunostaining results is given in Table 1 and Fig. 3.

**Table 1:** Cytokeratin 18 immunostaining in human tumors

	Entity	On TMA (n)	CK18 immunostaining					
			Analyzable (n)	Negative (%)	Weak (%)	Moderate (%)	Strong (%)	Positive (%)
Tumors of the skin	Pilomatrixoma	35	28	100.0	0.0	0.0	0.0	0.0
	Basal cell carcinoma	48	42	100.0	0.0	0.0	0.0	0.0
	Benign nevus	29	23	100.0	0.0	0.0	0.0	0.0
	Squamous cell carcinoma of the skin	50	44	93.2	6.8	0.0	0.0	6.8
	Malignant melanoma	48	43	100.0	0.0	0.0	0.0	0.0
	Merkel cell carcinoma	46	43	32.6	16.3	23.3	27.9	67.4
Tumors of the head and neck	Squamous cell carcinoma of the larynx	50	43	72.1	25.6	0.0	2.3	27.9
	Oral squamous cell carcinoma (floor of the mouth)	50	43	93.0	7.0	0.0	0.0	7.0
	Pleomorphic adenoma of the parotid gland	50	42	23.8	50.0	19.0	7.1	76.2
	Warthin tumor of the parotid gland	49	47	0.0	40.4	36.2	23.4	100.0
	Basal cell adenoma of the salivary gland	15	15	6.7	60.0	6.7	26.7	93.3
Tumors of the lung, pleura and thymus	Adenocarcinoma of the lung	200	176	0.0	29.5	28.4	42.0	100.0
	Squamous cell carcinoma of the lung	77	66	39.4	48.5	7.6	4.5	60.6
	Small cell carcinoma of the lung	20	16	18.8	12.5	50.0	18.8	81.3
	Malignant mesothelioma	48	40	12.5	12.5	15.0	60.0	87.5
	Mesothelioma, other types	28	18	11.1	33.3	16.7	38.9	88.9
	Mesothelioma, epitheloid	39	26	3.8	11.5	34.6	50.0	96.2
Tumors of the female genital tract	Squamous cell carcinoma of the vagina	48	20	90.0	5.0	0.0	5.0	10.0
	Squamous cell carcinoma of the vulva	50	33	100.0	0.0	0.0	0.0	0.0
	Squamous cell carcinoma of the cervix	50	35	88.6	2.9	0.0	8.6	11.4
	Adenocarcinoma of the cervix uteri	50	38	0.0	13.2	23.7	63.2	100.0
	Endometrioid endometrial carcinoma	236	212	0.5	14.2	22.2	63.2	99.5
	Endometrial serous carcinoma	82	53	1.9	15.1	34.0	43.4	92.5
	Carcinosarcoma of the uterus	48	38	21.1	26.3	34.2	18.4	78.9
	Endometrial clear cell carcinoma	8	7	0.0	57.1	42.9	0.0	100.0
	Endometrial carcinoma, low differentiated G3	13	12	33.3	33.3	25.0	8.3	66.7
	Endometrial stromal sarcoma	12	10	100.0	0.0	0.0	0.0	0.0
	Endometrioid carcinoma of the ovary	115	86	1.2	4.7	26.7	67.4	98.8
	Serous carcinoma of the ovary	567	495	0.8	13.7	31.9	53.5	99.2
	Mucinous carcinoma of the ovary	97	75	0.0	17.3	18.7	64.0	100.0
	Clear cell carcinoma of the ovary	54	47	8.5	34.0	31.9	25.5	91.5
	Carcinosarcoma of the ovary	47	41	22.0	14.6	24.4	39.0	78.0
	Brenner tumor	9	8	62.5	37.5	0.0	0.0	37.5
Tumors of the breast	Invasive breast carcinoma of no special type	1391	1001	3.6	21.8	23.5	50.9	96.2
	Lobular carcinoma of the breast	294	229	0.4	21.0	26.6	52.0	99.6
	Medullary carcinoma of the breast	26	22	59.1	18.2	9.1	13.6	40.9
	Tubular carcinoma of the breast	27	17	0.0	23.5	17.6	58.8	100.0
	Mucinous carcinoma of the breast	58	35	0.0	11.4	37.1	51.4	100.0
	Phyllodes tumor of the breast	50	32	0.0	37.5	21.9	40.6	100.0
Tumors of the digestive system	Adenomatous polyp, low-grade dysplasia	50	41	0.0	0.0	0.0	100.0	100.0
	Adenomatous polyp, high-grade dysplasia	50	44	0.0	0.0	2.3	97.7	100.0
	Adenocarcinoma of the colon	1932	1750	2.6	2.3	9.9	85.3	97.4
	Adenocarcinoma of the small intestine	10	5	0.0	0.0	0.0	100.0	100.0
	Gastric adenocarcinoma, diffuse type	226	161	5.6	32.3	28.6	33.5	94.4
	Gastric adenocarcinoma, intestinal type	224	156	7.1	23.7	19.2	50.0	92.9
	Gastric adenocarcinoma, mixed type	62	59	5.1	25.4	22.0	47.5	94.9
	Adenocarcinoma of the esophagus	133	70	1.4	12.9	7.1	78.6	98.6
	Squamous cell carcinoma of the esophagus	124	63	68.3	22.2	0.0	9.5	31.7
	Squamous cell carcinoma of the anal canal	50	33	75.8	15.2	9.1	0.0	24.2
	Cholangiocarcinoma	130	112	1.8	17.0	29.5	51.8	98.2
	Hepatocellular carcinoma	50	49	8.2	28.6	20.4	42.9	91.8
	Ductal adenocarcinoma of the pancreas	612	523	1.0	16.8	25.6	56.6	99.0
	Pancreatic/Ampullary adenocarcinoma	89	76	5.3	6.6	25.0	63.2	94.7
	Acinar cell carcinoma of the pancreas	13	12	0.0	0.0	16.7	83.3	100.0
	Gastrointestinal stromal tumor (GIST)	50	42	97.6	2.4	0.0	0.0	2.4
Tumors of the urinary system	Non-invasive papillary urothelial carcinoma, pTa G2 low grade	177	109	41.3	11.9	23.9	22.9	58.7
	Non-invasive papillary urothelial carcinoma, pTa G2 high grade	141	100	36.0	20.0	24.0	20.0	64.0
	Non-invasive papillary urothelial carcinoma, pTa G3	187	144	18.8	27.8	24.3	29.2	81.3
	Urothelial carcinoma, pT2-4 G3	1164	939	39.7	28.1	14.7	17.5	60.3
	Small cell neuroendocrine carcinoma of the bladder	18	18	33.3	33.3	11.1	22.2	66.7
	Sarcomatoid urothelial carcinoma	25	18	66.7	33.3	0.0	0.0	33.3
	Clear cell renal cell carcinoma	858	722	47.5	31.2	14.3	7.1	52.5
	Papillary renal cell carcinoma	255	209	2.4	15.3	15.8	66.0	97.1
	Chromophobe renal cell carcinoma	131	112	0.9	9.8	20.5	68.8	99.1
	Oncocytoma	177	144	1.4	11.8	16.7	69.4	97.9
	Clear cell (tubulo) papillary renal cell carcinoma	21	18	27.8	5.6	16.7	50.0	72.2
Tumors of the male genital organs	Adenocarcinoma of the prostate, Gleason 3 + 3	83	82	0.0	1.2	1.2	97.6	100.0
	Adenocarcinoma of the prostate, Gleason 4 + 4	80	73	0.0	4.1	1.4	94.5	100.0
	Adenocarcinoma of the prostate, Gleason 5 + 5	85	81	0.0	3.7	11.1	85.2	100.0
	Adenocarcinoma of the prostate (recurrence)	330	287	6.3	16.0	27.9	49.8	93.7
	Small cell neuroendocrine carcinoma of the prostate	17	16	25.0	18.8	37.5	18.8	75.0
	Seminoma	220	204	97.5	2.5	0.0	0.0	2.5
	Germ cell neoplasia in situ	85	67	64.2	29.9	3.0	3.0	35.8
	Embryonal carcinoma of the testis	50	46	0.0	23.9	19.6	56.5	100.0
	Yolk sack tumor	50	42	0.0	38.1	21.4	40.5	100.0
	Teratoma	50	27	70.4	14.8	0.0	14.8	29.6
Tumors of endocrine organs	Adenoma of the thyroid gland	114	109	0.0	5.5	35.8	58.7	100.0
	Papillary thyroid carcinoma	392	381	0.3	1.6	14.7	83.5	99.7
	Follicular thyroid carcinoma	158	150	0.0	2.0	34.0	64.0	100.0
	Medullary thyroid carcinoma	107	98	1.0	6.1	33.7	59.2	99.0
	Anaplastic thyroid carcinoma	45	43	41.9	32.6	14.0	11.6	58.1
	Adrenal cortical adenoma	50	36	91.7	5.6	0.0	2.8	8.3
	Adrenal cortical carcinoma	26	20	70.0	10.0	0.0	20.0	30.0
	Phaeochromocytoma	50	42	100.0	0.0	0.0	0.0	0.0
	Appendix, neuroendocrine tumor (NET)	22	15	0.0	8.3	0.0	91.7	100.0
	Colorectal, neuroendocrine tumor (NET)	10	10	0.0	0.0	10.0	90.0	100.0
	Ileum, neuroendocrine tumor (NET)	49	45	0.0	2.2	2.2	95.6	100.0
	Lung, neuroendocrine tumor (NET)	19	18	0.0	11.8	17.6	70.6	100.0
	Pancreas, neuroendocrine tumor (NET)	102	79	1.3	5.1	31.6	62.0	98.7
	Colorectal, neuroendocrine carcinoma (NEC)	11	9	33.3	22.2	22.2	22.2	66.7
	Gallbladder, neuroendocrine carcinoma (NEC)	4	4	50.0	0.0	25.0	25.0	50.0
	Pancreas, neuroendocrine carcinoma (NEC)	13	7	28.6	28.6	0.0	42.9	71.4
Tumors of haemotopoetic and lymphoid tissues	Hodgkin Lymphoma	45	39	100.0	0.0	0.0	0.0	0.0
	Non-Hodgkin Lymphoma	48	41	100.0	0.0	0.0	0.0	0.0
Tumors of soft tissue and bone	Tenosynovial giant cell tumor	45	43	100.0	0.0	0.0	0.0	0.0
	Granular cell tumor	53	32	100.0	0.0	0.0	0.0	0.0
	Leiomyoma	50	32	100.0	0.0	0.0	0.0	0.0
	Angiomyolipoma	91	74	98.6	1.4	0.0	0.0	1.4
	Angiosarcoma	73	55	87.3	7.3	3.6	1.8	12.7
	Dermatofibrosarcoma protuberans	21	16	100.0	0.0	0.0	0.0	0.0
	Ganglioneuroma	14	11	100.0	0.0	0.0	0.0	0.0
	Kaposi sarcoma	8	5	100.0	0.0	0.0	0.0	0.0
	Leiomyosarcoma	87	70	100.0	0.0	0.0	0.0	0.0
	Liposarcoma	132	98	100.0	0.0	0.0	0.0	0.0
	Malignant peripheral nerve sheath tumor (MPNST)	13	12	100.0	0.0	0.0	0.0	0.0
	Myofibrosarcoma	26	24	100.0	0.0	0.0	0.0	0.0
	Neurofibroma	117	103	100.0	0.0	0.0	0.0	0.0
	Sarcoma, not otherwise specified (NOS)	75	67	94.0	4.5	1.5	0.0	6.0
	Paraganglioma	41	37	100.0	0.0	0.0	0.0	0.0
	Primitive neuroectodermal tumor (PNET)	23	13	100.0	0.0	0.0	0.0	0.0
	Rhabdomyosarcoma	7	6	100.0	0.0	0.0	0.0	0.0
	Schwannoma	121	93	98.9	0.0	1.1	0.0	1.1
	Synovial sarcoma	12	10	100.0	0.0	0.0	0.0	0.0
	Osteosarcoma	43	29	96.6	3.4	0.0	0.0	3.4
	Chondrosarcoma	39	21	100.0	0.0	0.0	0.0	0.0

### CK18 expression, tumor phenotype, and prognosis

**Fig. 4:**
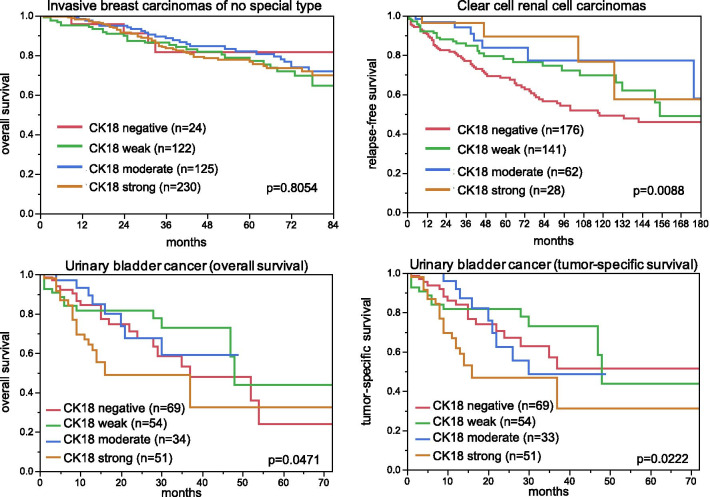
Cytokeratin 18 (CK18) immunostaining and patient prognosis. All bladder cancer patients had at least pT2 cancers and were treated by cystectomy

The relationship between CK18 expression and clinico-pathological data could be analyzed in two cancer types derived from CK18 positive precursor cells (breast and kidney cancer), one cancer type derived from epithelium with variable CK18 expression (urinary bladder) as well as in 230 squamous cell carcinomas of various organs of origin (n = 8), but all derived from squamous epithelia that are normally CK18 negative (Table 2, Fig. 4). Reduced or absent CK18 immunostaining was associated with high UICC stage (p = 0.0010), high Thoenes grade (p = 0.0086), advanced tumor stage (p < 0.0001), and poor prognosis in clear cell renal cell cancers (p = 0.0088) and with high grade and unfavorable molecular features such as ER/PR negativity (p < 0.0001 each)—but not with patient outcome—in invasive breast carcinomas of no special type. In squamous cell carcinomas, CK18 up-regulation was preferentially seen in cancers with advanced stage (13.2%/136 pT1-2 vs 27.7%/94 pT3-4; p = 0.0154), presence of lymph node metastasis (14.7%/95 pN0 vs 26.1%/92; p = 0.0354) and high grade (14.9%/134 G1-2 vs 28.0%/75 G3; p = 0.0767, data not shown). In bladder cancer, the CK18 expression pattern varied between subgroups. Within 1,353 patients that were treated by cystectomy, CK18 expression was unrelated to pathological parameters and patient outcome, however.

**Table 2:** Cytokeratin 18 immunostaining and tumor phenotype

	n	CK18 Immunostaining				p
		Negative (%)	Weak (%)	Moderate (%)	Strong (%)	
Invasive breast carcinoma of no special type						
All cancers	935	3.6	22.2	23.9	50.3	
pT1	497	4.2	18.9	25.0	51.9	0.0611
pT2	333	2.4	26.1	23.7	47.8	
pT3-4	73	6.9	19.2	16.4	57.5	
Grade 1	147	0.0	16.3	21.1	62.6	< 0.0001
Grade 2	479	2.1	19.0	26.7	52.2	
Grade 3	308	7.8	30.2	20.5	41.6	
pN0	439	3.2	20.1	23.9	52.9	0.6092
pN+	299	2.7	23.1	25.8	48.5	
HER2 negative	697	4.0	20.8	22.0	53.2	0.1689
HER2 positive	89	1.1	18.0	30.3	50.6	
ER negative	160	15.0	33.8	23.1	28.1	< 0.0001
ER positive	589	0.5	16.6	22.8	60.1	
PR negative	308	8.8	28.6	24.0	38.6	< 0.0001
PR positive	472	0.4	15.7	22.7	61.2	
Non-triple negative	619	0.8	17.5	22.5	59.3	< 0.0001
Triple negative	107	20.6	37.4	21.5	20.6	
Clear cell renal cell carcinoma						
All cancers	674	48.7	30.4	14.1	6.8	
ISUP 1	217	42.9	35.5	15.2	6.5	0.1312
ISUP 2	218	50.5	28.4	13.8	7.3	
ISUP 3	189	51.3	30.2	13.2	5.3	
ISUP 4	40	67.5	17.5	5.0	10.0	
Fuhrmann 1	32	37.5	34.4	21.9	6.3	0.2311
Fuhrmann 2	399	46.4	31.6	15.0	7.0	
Fuhrmann 3	194	52.1	30.9	11.3	5.7	
Fuhrmann 4	48	62.5	16.7	10.4	10.4	
Thoenes 1	239	41.0	33.9	17.6	7.5	0.0086
Thoenes 2	369	51.8	30.1	12.7	5.4	
Thoenes 3	65	60.0	20.0	7.7	12.3	
UICC 1	294	37.4	36.1	19.0	7.5	0.0010
UICC 2	34	50.0	32.4	11.8	5.9	
UICC 3	87	58.6	20.7	11.5	9.2	
UICC 4	70	57.1	34.3	7.1	1.4	
pT1	391	40.2	35.8	16.1	7.9	< 0.0001
pT2	72	59.7	25.0	11.1	4.2	
pT3	206	61.7	21.4	11.2	5.8	
pN0	114	55.3	28.9	7.9	7.9	0.5327
pN+	16	43.8	25.0	18.8	12.5	
pM0	102	39.2	35.3	15.7	9.8	< 0.0001
pM +	73	64.4	31.5	4.1	0.0	
Urinary bladder cancer						
All cancers	1353	37.0	25.4	18.0	19.7	
pTa G2 low	109	41.3	11.9	23.9	22.9	< 0.0001
pTa G2 high	100	36.0	20.0	24.0	20.0	
pTaG3	144	18.8	27.8	24.3	29.2	
pT ≥ 2 G3	921	39.9	28.0	14.8	17.4	
pT ≥ 2 G3 sarcomatoid	18	33.3	33.3	11.1	22.2	
pT ≥ 2 G3 small cell cancer	18	66.7	33.3	0.0	0.0	
pN0	293	36.5	26.6	16.7	20.1	0.2540
pN+	170	27.7	29.4	18.2	24.7

## Discussion

The standardized analysis of 11,952 cancers by IHC gives a comprehensive overview on CK18 staining in malignant tumors. The most valuable result of our study is a ranking order of CK18 positivity across a broad range of tumor entities which enables an estimate of the relative biologic importance of CK18 for individual tumor types and—together with the absolute numbers obtained in our analysis—a better assessment of the diagnostic impact of CK18 immunostaining results in specific diagnostic situations. The S-shaped curve of the CK18 expression frequencies found across 115 different tumor types reflects that frequent and intense CK18 immunostaining is commonly seen in cancers derived from CK18 positive normal cell types while most other tumors are often CK18 negative. 37 of 115 analyzed tumor entities (32.2%) showed CK18 positivity in > 97% of cases. Sporadic negative cases in the range of ≤ 3% in these cancer types may well be caused by technical issues. Some unexpected negative staining always occurs in TMAs because not all tissues are properly fixed in all areas (Tapia et al. 2004). Unequal immunostaining in tissues results in an immunostaining gradient across a tissue block and can result in false negative immunostaining, if TMA cores are taken from areas with poor reactivity (Fraune et al. 2020).

The group of cancers with variable CK18 immunostaining results including significant fractions of patients with CK18 positive and CK18 negative cancers, is heterogeneous in nature. This category contains cancer types arising from CK18 positive precursor cells but showing CK18 downregulation in a fraction of cases, tumor types arising from CK18 negative precursor cells but undergoing CK18 upregulation in a fraction of cancers, neoplasia’s derived from tissues with variable CK18 expression in benign precursors, and tumors with a mixed differentiation. The latter group contains tumors with a mixed glandular/squamous differentiation such as endometroid carcinomas of the uterus where adenomatous but not squamous epithelia stain positive as well as epithelial-mesenchymal tumors such as carcinosarcoma of the uterus and ovary, phyllodes tumor of the breast, teratoma of the testis or malignant mesothelioma. In these tumors, glandular epithelial but not mesenchymal tumor areas stain positive.

Cancers that markedly downregulate CK18 in a relevant fraction of cases include renal cell and breast cancers. True downregulation can easily be distinguished from artificial staining deficiency by presence of strongly staining normal cells in the same tissue spot. The analysis of larger cohorts of kidney and breast cancers for which clinical follow-up data were available identified significant associations of reduced CK18 immunostaining with unfavorable tumor phenotype and—in case of clear cell renal cell carcinoma—poor patient prognosis. These findings are consistent with earlier studies in breast cancer and may reflect a tendency towards a worse prognosis in cancer cells with an impaired cell differentiation (Woelfle et al. 2004; Willipinski-Stapelfeldt et al. 2005). That various cancers types that are by default poorly differentiated such as small cell carcinomas or anaplastic thyroid cancer showed lower CK18 positivity rates than their better differentiated counterparts is also consistent with the concept of a CK18 expression loss during tumor progression.

Squamous cell carcinomas are the best examples of epithelial tumors that are typically CK18 negative but can upregulate CK18. Even though CK18 immunostaining was not at all observed in any normal squamous epithelium samples from the lung, tonsil, skin, anal canal, oral cavity, or lip, a positive CK18 immunostaining was observed in 8 of 9 analyzed squamous cell carcinoma subtypes. That CK18 immunostaining was sometimes seen at high levels in these squamous cell carcinomas further demonstrates that these findings reflect true overexpression and not just a faint non-specific antibody binding. Our notion, that CK18 upregulation reflects aberrant differentiation or dedifferentiation in these cancers is supported by significant associations of elevated CK18 protein levels with high pT stage and presence of nodal metastasis that could be identified in a combined analysis of our 230 squamous cell carcinomas with available clinico-pathological data. These findings fit with data from several earlier studies suggesting a link between CK18 positivity and unfavorable clinico-pathological features and outcome in squamous cell carcinomas of the lung, esophagus, oral cavity, and pharynx (Makino et al. 2009; Broers et al. 1988; Nanda et al. 2012; Nhung et al. 1999; Safadi et al. 2019; Yang et al. 2018; Afrem et al. 2016).

The role of CK18 is less clear in tumor entities derived from tissues with variable CK18 expression such as in liver and urinary bladder cancer. In our analysis of 1353 urothelial carcinomas, 37% were completely negative and 20% of all cancers were considered strongly positive. That a marked difference in CK18 immunostaining was seen between pTa and pT2-4 urothelial carcinomas is consistent with the striking genomic differences between these tumor categories (summarized in (Knowles and Hurst 2015)). The absence of a statistically significant impact of CK18 expression on clinico-pathological features and outcome of pT2-4 carcinomas treated by cystectomy argues against a functional role of CK18 for cancer progression. CK18 plays a role in various cellular processes, such as securing the structure of the cytoplasm and mitochondria that are not directly related to cancer aggressiveness (Coulombe and Wong 2004). Considering the continuous increase of CK18 expression from basal and intermediate cells to the superficial and umbrella cells of the bladder epithelium, various levels of CK18 in cancer cells may also be related to the specific cell of origin.

Our data enable a comprehensive assessment of potential diagnostic applications of CK18 IHC. The close to 100% prevalence of CK18 expression in gastrointestinal cancers supports the concept of using CK18 measurement for metastasis detection (Oshima et al. 1996; Makino et al. 2009). The most useful diagnostic application of CK18 IHC may be the distinction of seminomas from other germ cell tumors of the testis. Only 12 of 204 analyzed seminomas (6%) but all of 88 embryonal carcinomas and yolk sack tumors of the testis showed CK18 expression. This finding is in line with data from an RNA and protein expression study identifying CK18 as one of the strongest discriminators of seminomatous versus non-seminomatous testicular germ cell tumors (Biermann et al. 2007). Pan-cytokeratin antibodies are often being included in diagnostic IHC panels to be used for the distinction of testicular cancer subtypes. In this context, cytokeratin positivity argues against seminoma which ideally should show either none or only weak cytokeratin staining. Considering that pan-cytokeratin staining is found in > 20% of seminomas and in > 80% of non-seminomas (summarized in (Emerson and Ulbright (2005))), the use of cytokeratin 18 showing positivity in 2.5% of seminomas and 100% of embryonal carcinoma of the testis as well as 100% of yolk sack tumors in our study might be preferable for testicular cancer subtyping. It appears conceivable that an antibody targeting just CK18 offers better specificity than an antibody targeting multiple cytokeratins.

**Fig. 5:**
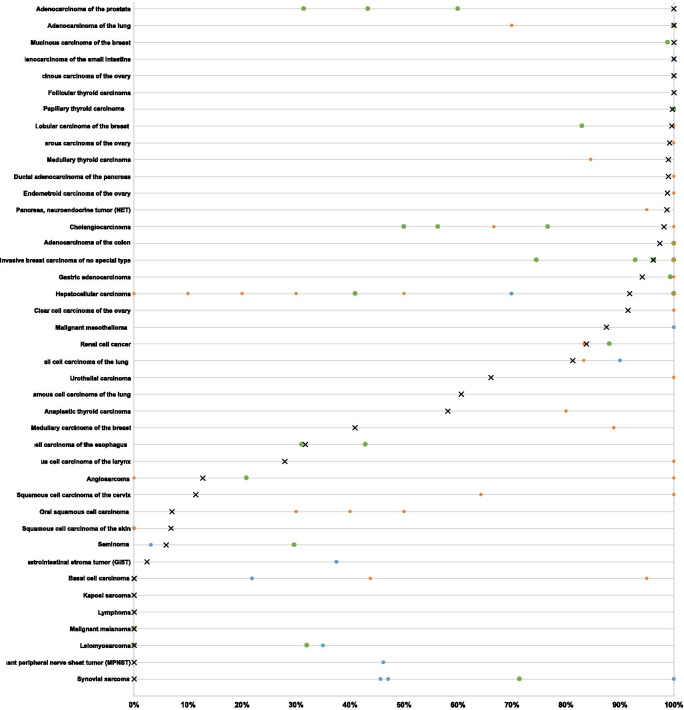
Graphical comparison of Cytokeratin 18 (CK18)  data from this study (x) in comparison with the previous literature. Orange dots are used for studies involving ≤ 20 cases, green dots are used for studies > 20 cases, blue dots are from Chu and Weiss 2002 (Review) (Chu and Weiss 2002). All studies are quoted in the list of references

Importantly, all prevalence’s described in this study are specific to the reagents and the protocol used in our laboratory. It is almost certain, that the use of different antibodies, protocols and interpretation criteria have jointly caused highly diverse literature data on CK18 expression in cancer (summarized in Fig. 5). It is well known, that different antibodies designed for the same target protein can vary to a large extent in their binding properties and that protocol modifications greatly impact the rate of immunostained cases (Saper 2009). However, the abundant data generated in this study would potentially make it possible to adjust a protocol for CK18 immunostaining and interpretation in a way that resulted in comparable frequencies of CK18 positivity. For that purpose, it might be sufficient to use smaller collections of tumors with high positivity rate such as adenocarcinomas of the prostate or the colorectum and of tumors with low positivity rates such as squamous cell carcinomas of various types to develop a protocol that results in comparable data as provided in this study.

## Conclusions

Our data show that CK18 is consistently expressed in various epithelial cancers, especially adenocarcinomas. Both loss of CK18 expression in cancers derived from CK18 positive precursor cells and neo-expression in malignancies derived from CK18 positive precursors tend to be linked to unfavorable tumor phenotype and disease outcome. Distinction of seminomas from other germ cell tumors of the testis appears to be the strongest diagnostic application of CK18 IHC.

## Data Availability

All data generated or analyzed during this study are included in this published article.

## Abbreviations

Akt1: Serine/threonine-protein kinase
CK18: Cytokeratin 18
ER: Estrogen receptor
ERK: Extracellular signal-regulated kinase
IHC: Immunohistochemistry
ISUP: International Society of Urological Pathology
MAPK: Mitogen-activated protein kinase
PR: Progesterone receptor
TMA: Tissue microarray
UICC: International Union Against Cancer
